# HIV diagnosis disclosure to infected children and adolescents; challenges of family caregivers in the Central Region of Ghana

**DOI:** 10.1186/s12887-018-1330-5

**Published:** 2018-11-22

**Authors:** Anna Hayfron-Benjamin, Dorcas Obiri-Yeboah, Stephen Ayisi-Addo, Peter Mate Siakwa, Sylvia Mupepi

**Affiliations:** 1 Department of Maternal and Child Health, School of Nursing and Midwifery, University of Cape Coast, Cape Coast, Ghana; 2 Department of Microbiology and Immunology, School of Medical Sciences, University of Cape Coast, Cape Coast, Ghana; 3 National AIDS/STI Control Program of the Ghana Health Service, Accra, Ghana; 4 Department of Basic Life Sciences, School of Nursing and Midwifery, University of Cape Coast, Cape Coast, Ghana; 5 Kirkhoff School of Nursing, Grand Valley State University, Michigan, USA

**Keywords:** HIV, Disclosure, Family caregivers, Children and adolescents, Ghana

## Abstract

**Background:**

Disclosure of Human Immunodeficiency Virus (HIV) to infected older children and adolescents is essential for both personal health maintenance and HIV prevention within the larger population. Non-disclosure of HIV status has been identified as one of the potential barriers to optimum adherence especially in children and adolescents. Like many other countries in the SSA region, Ghana has significant number of children and adolescents infected by HIV, who have increased survival times, due to increased access to ART. However, both family caregivers and healthcare workers face an array of challenges with the disclosure process, including the timing, what information about the child’s HIV status should be shared with him/her and how to go about it. The aim of the study was to identify family caregiver factors associated with non-disclosure of HIV status to infected children and adolescents accessing Antiretroviral Therapy (ART) at the three main ART sites within the Central Region of Ghana.

**Methods:**

A quantitative analytical survey was conducted among 103 family caregivers of HIV infected children (aged 6–17 years) assessing ART services in the Central Region of Ghana. Data were analyzed using SSPS version 21.

**Results:**

The age range of caregivers was 20–69 years. The study found a low disclosure rate (23.3%) among caregivers. Majority of the caregivers (80.6%) lacked knowledge on the process of disclosure (how and what to tell child), and majority (64%) also had never received guidance about the disclosure process from their healthcare providers. The main barriers to disclosure were caregiver lack of knowledge regarding the disclosure process and when to disclose, the fear of child’s reaction, and fear of stigmatization and associated negative social consequences.

**Conclusion:**

These findings suggest a lesser involvement of health care providers in preparing caregivers for the disclosure process. This therefore highlight the need for the National HIV/AIDS/STI Control Program to strengthen the involvement and training of healthcare providers in HIV diagnosis disclosure to infected children, based on context-specific policy guidelines informed by the WHO recommendations.

## Background

Disclosure of Human Immunodeficiency Virus (HIV) diagnosis to infected children is an important issue in clinical practice in recent times, because it presents with many clinical and psychosocial benefits that seek to improve the quality of life of people infected and affected by the HIV disease [1]. In the context of paediatric HIV, disclosure refers to a child gaining knowledge of his/her HIV status [2]. The American Academy of Paediatrics Committee (AAPC) and the WHO strongly recommend the disclosure of HIV diagnosis to older children of school age and beyond, on ethical and clinical grounds. Recognizing the importance in improved quality of long-term care for this vulnerable population, the WHO recommends that children of school age should be told their HIV positive status and that younger children should be told their status incrementally to accommodate their cognitive skills and emotional maturity, in preparation for full disclosure [3, 4].

Evidence from studies conducted in resourced countries, shows that informing children about their HIV diagnosis can have positive psychosocial and clinical outcomes. These include improved adherence with associated increased survival rates, improved personal health maintenance, decreased psychological effects associated with accidental disclosure and improved HIV prevention within the larger population [4–9]. Effective disclosure is so important because it is a start in meeting the often-repetitive education needs of HIV young people, around daily living with the virus and how it will influence decisions that they make in their social lives; including managing their own health, disclosing to significant others, and sexual choices [3, 5, 6, 10, 11]. UNESCO’s strategy for HIV and AIDS also reported that as these children grow older into adolescents, the knowledge about their disease will enable them make safe and healthy life choices about relationships, sex, and reproduction [5].

Several studies have evidently shown that children who are fully disclosed to, become self-motivated and are more likely to adhere to Antiretroviral Therapy (ART) and overcome external adherence challenges [6–9]. The success of ART leads to dramatic changes in the clinical course of HIV infection in paediatric patients and consequently increases their survival time. For ART to be successful, sustained and optimum adherence is required. On the other hand, non-disclosure of HIV status has been identified as one of the potential barriers to optimum adherence especially in children and adolescents [6, 12, 13]. Non-disclosure is linked to poor adherence, which would lead to treatment failure, increased viral load, increased risk of early disease progression or dramatic changes in the clinical course of HIV infection, and consequently decreased survival time of these young people. Also, if infected youth are non-adherent, they could potentially transmit drug-resistant virus to their sexual partners through unprotected sex, thereby increasing HIV spread [10–12].

In the early part of the epidemic, especially in the sub-Saharan Africa (SSA) where access to ART was limited, HIV/AIDS increasingly affected the health and welfare of infected children with more deaths recorded [3, 14]. Due to the very low survival rate, few providers were concerned about disclosing the diagnosis to these children [3]. However, the increased access to ART and its success in the treatment of paediatric HIV in recent times, has changed the face of the HIV epidemic in children, in such resource limited settings, and most children live longer than before [3, 15, 16]. This has therefore called for a change in the practice of non-disclosure and more caregivers must therefore be prepared to disclose to their infected children [10]. Contrary to this expectation, several studies have shown that the increased survival times has rather presented with one of the biggest psychosocial challenges that family caregivers face with regards to the disclosure of HIV diagnosis to their infected children [2, 13, 17, 18]. As such, more caregivers are hesitant or unable to disclose and many might choose to withhold an HIV diagnosis throughout the HIV-infected child’s life [1]. Researchers on the subject agree that the caregivers’ reluctance to disclose is especially so in the developing countries and that not only family caregivers are reluctant or find it difficult to disclose but healthcare workers as well [8, 9, 19, 20].

The disclosure situation is not different in Ghana. Enforcing adherence has resulted in confrontations and conflicts with caregivers since the caregivers are unwilling to explain to their infected children why they are on medications. Regardless of the hindrances to good adherence and improved self-care, some parents still have strong reservations for disclosure of status to their adolescent children, although their reasons are not well articulated [21]. Like many other countries in the SSA region, Ghana has significant number of children and adolescents infected by HIV, who have increase in survival times, due to increased access to ART [22]. Although the provision of ART is about a decade now, limited policy research on disclosure of HIV diagnosis to children has been carried out in the country.

This non-disclosure if allowed to continue would have negative ramifications for not only the affected adolescents, but the entire nation. This is because non-disclosure will lead to non-adherent and consequently poor treatment outcome such as treatment failures, increased drug resistance strains, increased viral load, and associated risk of HIV transmission to the general population [10–12].

It is for these reasons that understanding the influencing factors of disclosure of HIV status to infected children, is to be viewed as increasingly important in the management and care of HIV infected children. The study therefore sought to evaluate family caregiver factors that hinder the disclosure of HIV diagnosis to children in the Central Region of Ghana. The results of this study are critical in adding new knowledge that will facilitate training guidelines for health personnel curricula, directed at how the process of disclosure should be instituted. The findings of the study will also help bridge the existing gap in the literature about disclosure of HIV diagnosis to children in resourced limited settings.

## Methods

### Study site and population

A total of 103 family caregivers of children and adolescents aged 6–17 years on ART, who accompanied their children to assess HIV services at the three main ART sites within the Central Region, were sampled and included in the study. Literature suggests that the best age to disclose to a child is 6 years and above because at that age the child was able to understand disease and illness [2]. The definition of a child according to the children’s ACT 560, is a person below 18 years. The age group of children used in the study is based on these reasons.

The sites were: the Cape Coast Teaching Hospital, Cape Coast; St. Francis Xavier Catholic Hospital, Assin Fosu; and the Winneba Government Hospital, Winneba. These facilities offer both general medical care and antiretroviral treatment (ART) for all age groups including children and adolescents in the Central Region of Ghana.

A total of 110 registered dyads of caregiver and child that fell within the inclusion criteria were used. Out of the total number of the 110 registered dyads, six (6) were used as pilot and were not included in the main study; one (1) registered dyad had moved out of the region and could not be traced. Data were collected from the remaining 103. Fifty-eight (58) of the respondents were recruited from the Cape Coast Teaching Hospital, 23 from St. Francis Xavier Hospital, and 29 from the Winneba Government Hospital. Caregivers were recruited to participate as they waited for consultation and medication during their routine monthly visits for ART for their children.

### Study design and data collection procedure

A descriptive quantitative survey was conducted using a structured interviewer administered questionnaire. The validity and reliability of the data collection tools and procedures were also determined; Cronbach alpha was calculated as a measure of internal consistency for the total instrument and was found to have high internal consistency; Alpha co-efficients for the subscales ranged from 0.848 to 1.000. Data were collected between January and April, 2014. Two weeks prior to the main study a pilot study was conducted with six (6) caregivers of children on ART (2 each) from the study settings. Data collection was conducted during clinic attendance by the researcher and trained research assistants, who were all professional HIV counselors.

### Validity and reliability of the instrument

The validity and reliability of the data collection tools and procedures were determined; Cronbach alpha was calculated as a measure of internal consistency for the final instrument; the total instrument was found to have high internal consistency, with an alpha coefficient of 0.978 (Table 1). Alpha coefficients for the subscales ranged from 0.848 to 1.000. The scale used in assessing the knowledge had seven items and the Cronbach’s reliability coefficient alpha for this scale was 0.848. The scale used in assessing caregiver reasons for delayed disclosure had 10 items and the Cronbach’s reliability coefficient alpha for this scale was 1.000. The scale used in assessing non-disclosed caregiver reasons for non-disclosure had 21 items and the Cronbach’s reliability coefficient alpha for this scale was 1.000.

**Table 1:** Caregiver Characteristics by their Children’s HIV Disclosure Status (*N* = 103)

VARIABLE	Total n (%)	Disclosure rate	
		Disclosed =24 (23.3%) (*n* %)	Not-disclosed = *79 (76.7)* (n/%)
Gender of caregiver			
Male	18 (17.5)	8 (7.8)	10 (9.8)
Female	85 (82.5)	16 (15.5)	69 (67.5)
Age of caregiver (yrs.)			
20–39	44 (42.7)	7 (6.8)	37 (36.3)
40–59	48 (46.6)	7 (6.8)	41 (40**.**3)
60–69	11 (10.7)	10 (9.7	1 (0.1)
Marital status			
Married or cohabiting	46 (44.6)	9 (8.8)	37 (36.3)
Single	18 (17.5)	7 (6.8)	11 (10.8)
Divorced/separated/ widowed	39 (37.9)	8 (7.8)	31 (29.6)
Relation to child			
Biological mother	50 (48.5)	9 (8.8)	41 (39.8)
Biological father	9 (8.7)	3 (2.9)	6 (5.8)
Grandparents	19 (18.5)	7 (6.8)	12 (11.7)
Uncle or aunt/	20 (19.4)	5 (4.8)	15 (14.6)
Foster parents	3 (2.9)	0	3 (2.9)
Siblings	2 (1.9)	0	2 (1.9)
Financial situation			
Independent	75 (72.8)	22 (21.4)	53 (51.5)
Dependent	28 (27.3)	2 (1.9)	26 (25.2)
Level of education			
No formal education	24 (23.3)	3 (2.9)	21 (20.4)
Up to primary level	36 (34.9)	6 (5.8)	30 (29.1)
Up to secondary school level	34 (33.1)	8 (7.8)	26 (25.2)
Up to tertiary level	9 (8.7)	7 (6.8)	2 (1.9)
HIV status of caregiver			
Positive	56 (54.4)	13 (12.6)	42 (40. 8)
Negative	28 (27.2)	7 (6.8)	21 (20.4)
Don’t know	19 (18.4)	4 (3.9)	16 (15.5)
Child’s age at disclosure			
6–10	49 (47.6)	1 (1.0)	48 (46.5)
11–14	40 (38.8)	9 (8.7)	31 (30.1)
> 14	14 (13.6)	14 (13.6)	0 (0)

### Data analysis

Data were cleaned, coded and captured on Microsoft Excel and analyzed using statistical packaging for social sciences (SPSS) software version 21. Summary statistics was used to calculate and interpret the mean, and range of continuous variables under investigation and to obtain frequency tables for discrete variables. The data was presented through tables and frequency distributions. Inferential statistics, specifically factor analysis was also performed on the non-disclosed caregivers reasons for non- disclosure, to determine the most important factors that hindered the disclosure process.

### Ethical considerations

The University of Cape Coast institutional project review board (UCCIRB) granted ethical approval for the study before commencement. Clearance was also given by the NACP/GHS, after signing a data sharing agreement. Permission was also gained from management of the three hospitals, where the study was conducted. Confidentiality was ensured at all stages of the process. Data collection was preceded by an informed consent signed by participants.

## Results

Data were collected from a total of = 103 of disclosed and non-disclosed caregivers of children aged between 6 and 17 years. All completed the questionnaire adequately making the response rate 100%. As shown in Table 1, the caregivers aged between 20 and 69 years with a mean age of 42 years. The highest proportions of the caregivers were females and the biological mothers of the children (*n* = 85, 82.5%) and (*n* = 50, 48.6%), respectively. Biological fathers constituted less than a tenth (*n* = 9, 8.7%) More than half (*n* = 56, 54.4%) of the caregivers were HIV positive and almost one fifth (*n* = 19, 18.4%) did not know their HIV status. The highest proportion (*n* = 44, 42.7%) were married. With regards to their educational attainment, the majority (*n* = 60, 58.3%) comprised those with either no formal education or had up to primary level, and less than a tenth 9 (8.7%) had completed tertiary education. The majority (*n* = 75, 72.8%) also reported being financially independent.

Comparing the disclosed and non-disclosed groups, less than a tenth (*n* = 9, 8. 8%), of the 50 biological mothers interviewed had disclosed to their children as against the greater proportion (*n* = 41, 39.8%) who had not. The biological mothers also constituted greatest proportion (*n* = 41, 39.8%) out of the total 79 non-disclosed caregiver category. Also, majority (*n* = 42, 40.8%) of the non-disclosed caregivers were HIV positive themselves. It is noteworthy that, a small proportion (*n* = 2, 1.9%) of caregivers who had attained tertiary education had not disclosed as compared to highest proportion (*n* = 51, 49.5%) of those with primary or no educational background.

Table 2 presents the disclosed caregivers disclosure related experiences. Majority (*n* = 16, 66.7%) were able to disclose with the support of their healthcare providers with only a third (*n* = 8, 33.3%) being able to do so by themselves. Interestingly, only a quarter were able to disclose confidently. Regarding the time frame for disclosure after child’s diagnosis, only a fifth (*n* = 5, 20.8%) out of the 24 caregivers who have disclosed to their children were able to disclose within a year. It took the majority (*n* = 10, 41.7%) between 1 and 5 years, and more than a quarter (*n* = 9, 37.5%) after 5 years. Disclosed caregivers reported that they delayed the disclosure for various reasons. For the disclosed group, the main reasons for the disclosure were child being of age and pubertal age (*n* = 69, 87.3%) and support from healthcare providers (*n* = 59, 74.7%). More than half (*n* = 57, 72.2%) and (*n* = 56, 70.9%) respectively, had to disclose because their children were becoming increasingly curious about their daily medications and their routine clinic attendance.

**Table 2:** Disclosed Child and Caregiver Disclosure Related Experience (*n* = 24)

Variable	*n* (%), Mean
Child’s Age at disclosure (years)	
6–10	1 (4.2)
11–14	9 (37.5)
>14	14 (58.3)
Mean	10.4
Time frame of disclosure after child’s diagnosis	
<1 year	5 (20.8%)
1–5 years	10 (41.7%)
> 5 years	9 (37.5%)
Caregiver supported by health provider in disclosing	
Yes	16 (66.7)
No	8 (33.3)
Caregiver level of confidence at disclosing	
Confident	6 (25.0)
Somehow confident	10 (41.7)
Not at all confident	8 (33.3)
Reasons for the disclosure^a^	
Child has reached puberty and is of age	69 (87.3)
Child increasingly becoming curious about daily medications	57 (72.2)
Child inquisitiveness about routine clinic attendance	56 (70.9)
Child has started talking about sex and sexual relationship	54 (68.4)
My healthcare provider asked me to disclose and offered Support	59 (74.7)

^a^ *multiple responses*

**Fig. 1:**
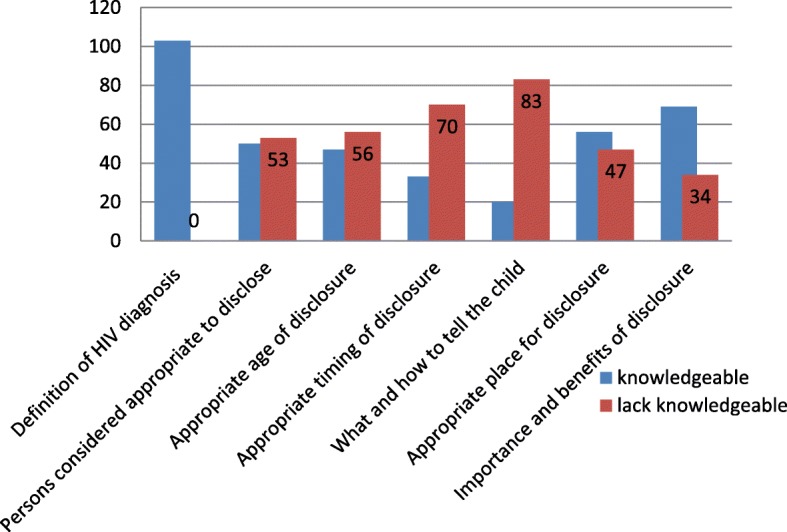
Caregivers level of knowledge regarding HIV diagnosis disclosure to HIV infected children (N = 103)

The level of knowledge of the respondents regarding the definition, benefits and the process of HIV diagnosis disclosure to an infected child were assessed and the data presented in Fig 1. Each item had more than one expected response and participants who were able to score more than half (50%), were graded knowledgeable, whilst those who scored less than 50% were graded as lacking knowledge. The expected responses were generated from the WHO guideline for disclosing the HIV diagnosis to infected children. The Cronbach’s reliability coefficient alpha for this 7 items scale was 0.848.

All caregivers (*n* = 103, 100%) could define HIV diagnosis disclosure and majority (*n* = 56, 54.4%) had knowledge about the appropriate places where disclosure can take place. Majority (*n* = 69, 67.0%) were also knowledgeable about the benefits or importance of disclosure. On the other hand, more than 50% of the respondents lacked knowledge regarding persons considered appropriate to disclose and appropriate child’s age of disclosure process. Concerning appropriate timing for the disclosure more than two thirds (*n* = 70, 68.0%) lacked knowledge. What and how to tell child was the biggest hindrance of which (*n* = 83, 80.6%) lacked knowledge.

To help give better explanations to the outcome of the knowledge level assessment, caregivers were also asked if they had received any form of counseling or guidance as to how to disclose. Out of the 103 respondents, only about a third (*n* = 35, 34.0%) had been taught by their healthcare providers regarding how to disclose the HIV diagnosis to their infected children, whilst the majority (66.0%) had not been taught how to do so. The other 2 (1.9%) of the caregivers also learned how to disclose from their peer educators.

### Caregiver barriers towards HIV diagnosis disclosure to their infected children

Caregivers completed a survey focused on reasons for delayed or non-disclosure. Although there are some caregivers who have disclosed, as described above, most of them delayed disclosure. Two barrier scales were used respectively for the caregivers who delayed disclosure and those who have not disclosed. Scale 1 contained 10 items (Table 3) whilst the main barrier scale comprised 21 items (Table 5).

**Table 3:** Disclosed Caregivers Reasons for Delayed Disclosure (*N* = 20)

Variable	Agree, *n* (%)
I did not know how the child would react	19 (95.0)
The child was too young and not mature enough to know	18 (90.0)
Disclosure to child at that age would not have made any difference in child’s treatment/care	11 (55.0)
I did not know how to tell the child about this diagnosis	14 (70.0)
I did not know what to tell child	14 (70.0)
I wanted to protect child from social stigma and discrimination	16 (80.0)
I was afraid others would get to know if child fails to keep the information secret	16 (80.0)
I was afraid disclosure would cause child to be unhappy/depressed	15 (75.0)
I was afraid about child asking me how one became infected, because child might blame me.	14 (70.0)
I could not have discussed sex with child at that age if child asked me to explain how he/she got infected.	18 (90.0)

### Disclosed caregivers’ reasons for delayed disclosure

Table 3 presents caregiver reasons for delaying in disclosing to their infected children and it revealed that more than 55% agreed to all the factors as reasons for the delayed disclosure. The most important barriers to timely disclosure identified in terms of ranking were; fear of how child would react to the news of the diagnosis (95%), child not being old enough to understand (90%), lack of caregiver readiness to discuss sex if child ask how they got infected (90%), fear of child not being able to keep diagnosis a secret and letting others know (80%), and protecting child from HIV diagnosis related stigma and discrimination (80%). A significant proportion (*n* = 14, 70%) of caregivers also delayed the disclosure because they did not know what and how to tell child. Worthy of note, more than half (*n* = 11, 55%) of the caregivers felt that disclosure to child at that age would not have made any difference in child’s treatment/care. This implies a significant number of the caregivers believe that disclosure at a certain age, has some impact on the child’s treatment or care.

### Non-disclosed caregivers reasons for non-disclosure

**Fig. 2:**
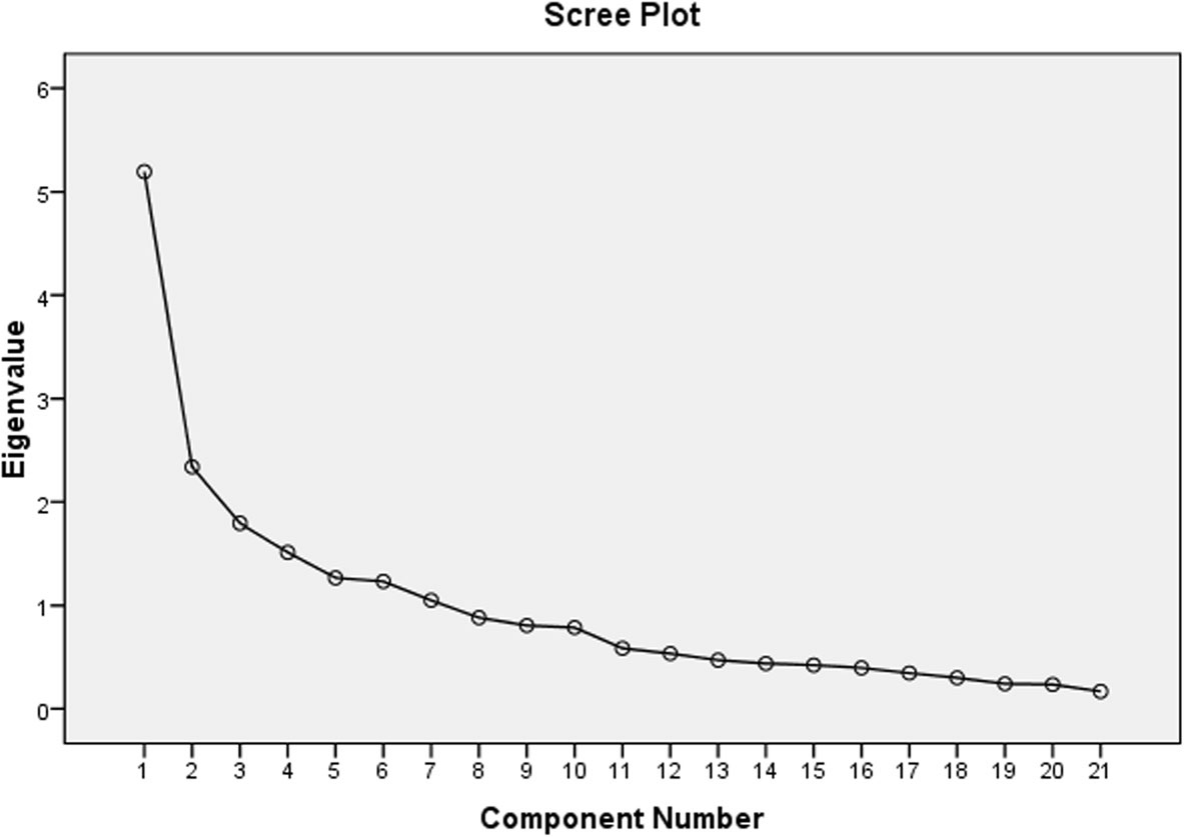
Scree plot for factor analysis of the barriers to disclosure

For the non-disclosed caregiver reasons for non-disclosure, a factor analysis was performed with a 21 items barrier scale to identify and label the main caregiver barriers to disclosure. Principal component factor analysis of the 21 item-instrument was performed. After this had been applied, the new factors represented linear combinations of variables with significant eigenvalues. Tables 4 and 5 respectively, present the total variance explained and the barriers hindering respondents, whilst Fig 2 is the scree plot of the eigenvalues. The factor analysis yielded a seven-factor solution with an explained variance of 68.6%, which had eigenvalues greater than 1.00. Since all seven (7) factors had eigenvalues greater than 1, the final factor solution represented 68.6% of the variance in the data. According to the scree plot (Fig 2), the slope of the curve became emergent at the seventh point, as such a seven-factor instrument was decided upon.

**Table 4:** Total Variance Explained for Barriers to the Disclosure Process

Component	Initial Eigenvalues			Extraction Sums of Squared Loadings		
	Total	% of Variance	Cumula-tive %	Total	% of Variance	Cumula-tive %
1. Caregiver lack of knowledge	5.196	24.745	24.745	5.196	24.745	24.745
2. Caregiver fear of stigmatization	2.338	11.132	35.877	2.338	11.132	35.877
3. Protection of child from hurt and social rejection	1.795	8.546	44.422	1.795	8.546	44.422
4. Discouragement from family, friends, and previous disclosure attempts	1.515	7.217	51.639	1.515	7.217	51.639
5. Fear of losing family	1.268	6.038	57.677	1.268	6.038	57.677
6. Fear of child’s reaction to the diagnosis	1.234	5.876	63.553	1.234	5.876	63.553
7. Untimeliness in terms of child’s age or maturity.	1.050	5.002	68.555	1.050	5.002	68.555

**Table 5:** Caregivers barrier to the disclosure process

	Barriers						
	Lack of Education	Fear of stigmatization	Fear of hurting child	Discouragement	Disrupting child’s education	Fear of divorce	Untimeliness
My care provider did not teach me ho*w*/what to tell child	0.807						
I do not know the exact age to disclose	0.723						
I don’t know how child will react and how to manage such reactions	0.645						
I feel guilty/ashamed for transmitting the infection	0.635						
I do not know how to explain sex to child if asked how he/she got infected	0.498						
I don’t know how my religious members will think of me		0.768					
I may lose my job if others get to know through my child		0.733					
I am afraid to lose my social status		0.699					
I have witnessed families who face stigma and discrimination because they disclosed.		0.628					
Protect the child from depressing information.			0.777				
Fear of rejection by the child			0.727				
I am afraid the child might not be able to keep the diagnosis a secret leading to social rejection			0.657				
My family discouraged me from disclosing				0.778			
In the past I tried to disclose but I failed				0.706			
My friends discouraged me from disclosing				0.572			
My in-laws will distrust me and I will lose my marriage if through child others get to know					0.842		
My family will disown me should they hear of my child’s diagnosis					0.642		
Disclosure will cause lack of concentration in school						0.779	
Disclosure will decrease child’s quality of life						0.456	
Child is too young to know or understand							0.753
Disclosing to children before their teen years will not make any difference to the child							0.535

The remaining 14 items were entered into the factor analysis. All items were loaded on expected factors, and the variance was more than 0.45 for most items; with only eight (8) below 0.4 (Table 5).

Table 5 shows the seven barriers to disclosure that emerged in order of ranking. Of the seven factors, the two most important identified were the lack of knowledge and fear of stigmatization. Those two factors had eigenvalues greater than 2 and had accounted for 24.74 and 11.13% of the total variation in the data, respectively. This means that the two are the most disturbing barriers. The remaining five items all had eigenvalues of less than 2.00 but more than 1.00.

The strongest factor 1 as shown in Table 5 is lack of knowledge on the disclosure process. Item loading on this factor included five items: “my caregiver did not teach me how to disclose”, “I do not know what and how to tell”, “I do not know how child will react and how to handle any negative reaction”, “I do not know the exact age at which to tell the child.”, and “I do not know how to explain sex to child if asked how he/she got infected”. Item loading on the next disturbing factor, fear of stigma also included four items: “I do not know how my religious members will think about me”, “I may lose my job if through child others get to know”, “I am afraid to lose my social status”, and “I have witnessed families who face stigma and discrimination because they disclosed. I don’t want my family to go through that stress.”

The highest of the remaining five factors is caregiver fear of hurting/harming child, which had three specific item loading: “Protect the child from depressing information. The child deserves a happy childhood”, “fear of rejection by the child”, and “afraid the child might not be able to keep the diagnosis a secret which may lead to such as social rejection”. The lowest of the factors was that of untimeliness and the specific item loading include two items; “child is too young to know” and “disclosing to children before their teen years will not make any difference to the child”.

## Discussion

The study revealed that a greater proportion (*n* = 79, 76.7%) of the children had not been told of their HIV diagnosis by their caregivers. These findings are consistent with those from other studies [13, 19–21, 23]. Although the documented prevalence of disclosure of HIV diagnosis to infected in children from well-resourced countries vary widely, from 18 to 77% [2, 23], in resource limited countries the prevalence remains low. Data from a previous Ghanaian study and two South African studies reported low levels of disclosure of HIV diagnosis to children on antiretroviral therapy [13, 23, 24]. In a cross-sectional study on the prevalence and determinants of disclosure among caregiver-child dyads from the Pediatric HIV/AIDS Care Program at Korle-Bu Teaching Hospital (Accra, Ghana), Kallem and colleagues reported prevalence of 21% [13]. In addition, clinical observation with HIV-positive children at three paediatric HIV clinics in a tertiary hospital in Johannesburg suggested that disclosure of HIV status rarely occurs [23]. Similar observation has been made in one of the largest ART sites in Ghana, the Komfo Anokye Teaching Hospital [21].

More than half (54.4%) of the caregivers were HIV positive with less than a fifth (18.4%) who did not know their HIV status and less than a tenth (*n* = 9, 8. 8%), of the 50 biological mothers interviewed had disclosed to their children whilst biological mothers constituted greatest proportion (*n* = 41, 39.8%) of the total non-disclosed caregiver category. It is worth noting that, majority (*n* = 42, 40.8%) of the non-disclosed caregivers were HIV positive themselves. The findings confirmed what has been reported in several other studies that parents’ own HIV status and fear of being blamed by the child can influence their decision-making regarding disclosure of the child’s HIV status as well as their ability to disclose [10, 13, 25, 26]. One plausible reason given to this disclosure pattern is that most of HIV positive parents feel guilty and responsible for infecting the children and therefore, failed to disclose in order to protect themselves from their child’s reaction, anger or blame on learning about the caregivers’ own HIV diagnosis [8, 9, 19, 20]. Furthermore, due to the stigma associated with the disease as well as the sexual mode of transmission, disclosure has been shown to be most difficult, especially, for biological parents who might be particularly worried about their children learning of their illness [15, 17, 19, 20, 27]. It is therefore not surprising that, caregivers who disclosed early tend to be non-biological parents of the infected children and likewise most children who knew their diagnosis were those living with non-biological caregivers [13, 15, 20]. Another observation made from this study is that, although a small proportion, majority of caregivers who had attained tertiary education had disclosed as compared to those with primary or no educational background. Possibly, this is because, the educated feel more equipped to handle the disclosure process than the uneducated. Although similar studies conducted in Ethiopia, Thailand and South Africa, have cited caregiver educational background as a correlate of disclosure to HIV-infected children, our findings are contrary to theirs. Those studies reported a higher rate of disclosure among caregivers with a lower level of education [19, 28].

Low disclosure rates in this study could also be attributed to family caregiver lack of disclosure related knowledge and skills as well as the low involvement of health care providers in disclosing the HIV diagnosis to their infected children. Caregiver knowledge assessment regarding the disclosure process showed that all (100%) caregivers in this current study were aware of the concept HIV diagnosis disclosure and knew that at a point in the child’s life they needed to disclose the HIV diagnosis to the child. Although generally, majority (67.0%) had good knowledge about the importance of disclosure, it is worth noting that majority lacked knowledge about the disclosure related process: Greater proportion (80.6%) of the caregivers lacked knowledge about what to tell child and how to go about it. Majority (more than 50%) also lacked knowledge about the appropriate person to disclose to child, appropriate timing, and appropriate child’s age, for the disclosure.

Caregiver lack of knowledge could be attributed to the poor preparation and lack of teaching by their respective healthcare providers. In our study, about two thirds (66%) of the caregivers, had not been taught or received any guidance on how to disclose to their children. The poor preparation could also explain why only a small proportion (25%) of the disclosed caregivers was reported of being confident at disclosing and also why a greater proportion (66.7%) were only able to disclose with the support of a healthcare provider. This data was comparable with findings from previous studies which indicated that low levels of direct involvement of health care professionals in disclosure process was associated with low prevalence of disclosure among family caregivers [19, 23, 29]. The findings suggest that health care providers played a major role in initiating disclosure with children in clinical settings.

From the above findings and discussions, it is therefore not surprising that, caregiver’s lack of knowledge on the process of disclosure was the most distinct barrier identified in this study, Specifically, caregivers lacked knowledge about when, how and what to tell the child, how to explain the infectious process to the child, and how to manage any negative child reaction with regards to the disclosure. A number of studies have reported family caregivers’ lack of knowledge regarding the appropriate age, timing, and what to tell as being linked to the delay in diagnosis disclosure to infected children [6, 24, 26, 30].

Caregiver fear of disclosure associated stigmatization and consequences such as social rejection were the second highest ranking barrier identified in this study. This was closely followed by caregiver fear of child being hurt or harmed as a consequence of negative social reaction towards a positive HIV status. The four other significant barriers identified namely; untimeliness with regards to child’s age; discouragement from family members, friends, and previous disclosure related negative experience; fear of divorce; and interrupting child education, were observed to be all closely linked to caregivers’ fear of negative social consequences associated with stigma and discrimination, should the child fail to keep the diagnosis a secret and others get to know about child’s HIV status. Protecting the child from the stigma associated with HIV has been documented as one of the major reasons for non-disclosure of HIV diagnosis to children [23, 25, 31, 32]. Other studies reported that caregivers often delayed the disclosure because of caregiver fears that the younger child would tell others and face discrimination.

This study also found untimeliness with regards to child’s age as one of the barriers. Majority of caregivers in this current study, delayed disclosure because they believed that the child was too young or immature to understand the disease. The findings are similar to those reported in other studies [6, 24, 25, 27, 32]. Child’s age is also closely linked to ability or inability to keep the diagnosis a secret, and over the years this keeps appearing in studies on disclosure of HIV diagnosis to children. Caregivers are reluctant to disclose the HIV diagnosis to their younger children because they perceived that at that young age, children lack the emotional and cognitive maturity needed to fully comprehend the disease and its implications, or to cope with the diagnosis [6, 18, 32].

This barrier could explain why in this current study, majority (*n* = 79, 76.7%) of the children had not been told of their HIV diagnosis, and why almost all (99%) of the 49 children that were below 10 years of age had not been disclosed to, whereas all of those above 14 years had been disclosed to. The disclosure related age distribution pattern implies that children below 10 years were least likely to be disclosed to as compared to those above 14 years. These findings are congruent with results from other studies from various parts of the world. Majority of caregivers in those studies felt that the child should be told about their HIV diagnosis around 14 years. Caregivers are more likely to disclose the HIV diagnosis to children over 13 years for varied reasons. The main reason identified are that older children have emotional maturity and intellectual capacityand are considered mature enough to cope with the news of their HIV status, and are also able to understand concepts of health, disease, and more complex concepts of chronic illness. Also, its because sex education and prevention of the spread of infection may require disclosure [11, 19, 23–25, 32].

The study’s main limitation was in terms of the generalizability of its results. All participants were recruited from the Central Region of Ghana, as such, the study cannot be generalized to caregivers outside the Central Region of Ghana, due to differences in cultural practices. Further studies are therefore needed to determine whether the findings are representative of the situation in other areas of Ghana.

## Conclusion

This study reiterates the findings of other studies from the SSA region that caregiver lack of knowledge regarding the disclosure process greatly hinders timely disclosure of the HIV diagnosis to infected children. The fear of stigma and associated negative social consequences such as, social rejection and loss of social status also greatly influenced the disclosure process. Stigma and discrimination remain a threat to timely disclosure and if not well addressed, the nation would be faced with majority of adolescents who are not disclosed to. This could result in associated negative consequences such as non-adherence to treatment, increased viral load, and increased risk of HIV transmission among the youth. There is therefore the need for healthcare providers and other stakeholders to continue relentlessly in the campaign against HIV related stigma and discrimination.

The findings also suggest a lesser involvement of health care providers in preparing the caregivers for the disclosure process, indicating weaknesses in the psychosocial aspect of HIV management of children and adolescents. The findings highlight the need for the NACP to strengthen healthcare providers’ involvement in HIV disclosure to children and adolescents. This would require development of context-specific  policy guidelines informed by the WHO recommendations on disclosure to children and adolescents. Such guideline will serve  as a resource for healthcare providers to provide standardized training to the family caregivers. Further studies are required to explore healthcare provider’s knowledge, attitude, and practices of the disclosure process. This would help identify gaps in the healthcare system and inform policy makers and stakeholders on how to tackle the problem.

## Abbreviations

AIDS: Acquired immune deficiency syndrome
ART: Antiretroviral therapy
ARVs: Antiretroviral drugs
HIV: Human immunodeficiency virus
NACP: National AIDS/STI Control Programme
OI: Opportunistic infection
PLHIV: People living with HIV
PMTCT: Prevention of Mother-To-Child Transmission
UNAIDS: The Joint United Nations Programme on HIV/AIDS
UNESCO: United Nations Education, Scientific, and Cultural Organization
UNICEF: The United Nations Children Fund
USAID: United States Agency for International Development
WHO: World Health Organization
